# Determining Relative Dynamic Stability of Cell States Using Boolean Network Model

**DOI:** 10.1038/s41598-018-30544-0

**Published:** 2018-08-13

**Authors:** Jae Il Joo, Joseph X. Zhou, Sui Huang, Kwang-Hyun Cho

**Affiliations:** 1 Department of Bio and Brain Engineering, Korea Advanced Institute of Science and Technology (KAIST), Daejeon, 34141 Republic of Korea; 2 Institute for Systems Biology, Seattle, WA USA; 3 Kavli Institute for Theoretical Physics, UC Santa Barbara, California, USA

## Abstract

Cell state transition is at the core of biological processes in metazoan, which includes cell differentiation, epithelial-to-mesenchymal transition (EMT) and cell reprogramming. In these cases, it is important to understand the molecular mechanism of cellular stability and how the transitions happen between different cell states, which is controlled by a gene regulatory network (GRN) hard-wired in the genome. Here we use Boolean modeling of GRN to study the cell state transition of EMT and systematically compare four available methods to calculate the cellular stability of three cell states in EMT in both normal and genetically mutated cases. The results produced from four methods generally agree but do not totally agree with each other. We show that distribution of one-degree neighborhood of cell states, which are the nearest states by Hamming distance, causes the difference among the methods. From that, we propose a new method based on one-degree neighborhood, which is the simplest one and agrees with other methods to estimate the cellular stability in all scenarios of our EMT model. This new method will help the researchers in the field of cell differentiation and cell reprogramming to calculate cellular stability using Boolean model, and then rationally design their experimental protocols to manipulate the cell state transition.

## Introduction

Cell state transition is at the core of many cell biology processes in metazoan, such as cell differentiation, cell stress response, epithelial-to-mesenchymal transition (EMT) in development, but also in artificial cell type reprogramming, such as generation of iPSC (induced pluripotent stem cell) from differentiated cells or directed differentiation of multipotent cells, including stem cells and iPSC, into specialized cell types^1–4^. Cell state transitions can be experimentally induced by ectopic control of the activity of key regulatory genes or by providing the appropriate environmental signals^5–8^. The conventional approach to identify these molecular levers to trigger the desired state transitions has been to use an educated guess based on known functions of key regulators or of relevant regulatory pathways and typically involves trial and error (such as high-throughput screening in the extreme case^9^). However, subsequent applications of the identified regulators, e.g. overexpressing or suppressing of a particular set of genes, and the use of empirical cytokine cocktails^10^, usually achieve low efficiency and necessitate selection of cells. The latter is further compromised by induction of undesired transitions, e.g. of stem cells into not only the intended lineage, but into “wrong” neighboring lineages^11–14^. For example, generation of iPSC from differentiated cells (“reprogramming”) has an efficiency that is usually below 1% when using the classic Yamanaka protocol^15^, i.e. 1% of cells reach the desired destination state, the iPSC. Similarly, the efficiency of reprogramming pancreatic exocrine cells to beta cells using a set of three TFs is below 25%^7^. In reprogramming human embryonic stem cells human to retinal photoreceptor cells, less than 20% of cells at each step differentiated to desired cell types^5,6^. The low efficiency of directed (de)differentiation is in line with the observation that the cells of the clonal cell population are heterogeneous^16^, suggesting the possibility that they may respond in distinct manners to the inducing signal applied.

The rationale for reprogramming protocols being derived from pathway diagrams involving “fate-determining” master regulators embodies linear causation and only allows for qualitative deterministic predictions of the fate outcome of manipulations. Such information on functions of regulatory factors and pathways cannot take into consideration the dynamics, including the ubiquitous stochastic fluctuations of gene activities^17^; hence it does not allow prediction of a graded response, such as the submaximal efficiency (≪100%) of desired state transitions. The pathway paradigm also cannot predict the pleiotropic and stochastic effects that lead to the diversification of cells into several lineages in response to the reprogramming perturbation. Here we propose a theoretical framework that goes beyond deterministic linear causation of regulatory pathways and takes into account entire gene regulatory network (GRN) and their dynamics. The latter refers to how GRN imposes constraints on the collective change of gene expression (since gene activities are coupled across all the genes) and determines trajectories of cell states, thereby affording robustness to cell states by constraining some of the random fluctuations, while still allowing cells to escape the constraints by the GRN. This systems biological approach is not only grounded on fundamental principles of the theory of dynamical systems but also offers guidance for the design of cell reprogramming protocols that can help understand the stochastic diversification of cell response to regulatory factor manipulations and can be used to improve efficiency of reprogramming into the desired state.

In brief, any observable cell state, such as a cell type, manifests a characteristic genome-wide gene expression pattern which allows the expressed genes to perform specific biological functions and to implement the characteristic phenotype of a cell. We use a vector ${\boldsymbol{x}}=({x}_{1},\,{x}_{2},\cdots ,{x}_{n})$ to represent the gene expression pattern consisting of gene expression values (“gene activities”) *x*_*i*_ of the *n* relevant genes associated with the state of a cell. Gene expression patterns arise because the individual gene activities are coordinated by the gene regulatory network. As a consequence of the gene regulatory interactions, each gene *x*_*i*_ can only change its value as a function of all the genes in the GRN or a particular subset; hence ${\dot{x}}_{i}={F}_{i}({x}_{1},\,{x}_{2},\cdots ,{x}_{n})$, or for the entire system of the *n* genes in the network $\dot{{\boldsymbol{x}}}={\boldsymbol{F}}({x}_{1},\,{x}_{2},\cdots ,{x}_{n})$ which is an *n*-dimensional system of ODEs (ordinary differential equations). An attractor x*, a stable fixed point or a limit cycle, corresponds to a distinct nominal cell state^18^, such as an embryonic stem cell, a liver cell, a stressed cell, etc., defined by its characteristic gene expression profile. In physics, x* is a non-equilibrium stable steady-state in the GRN as an open thermodynamic system.

**Figure 1:**
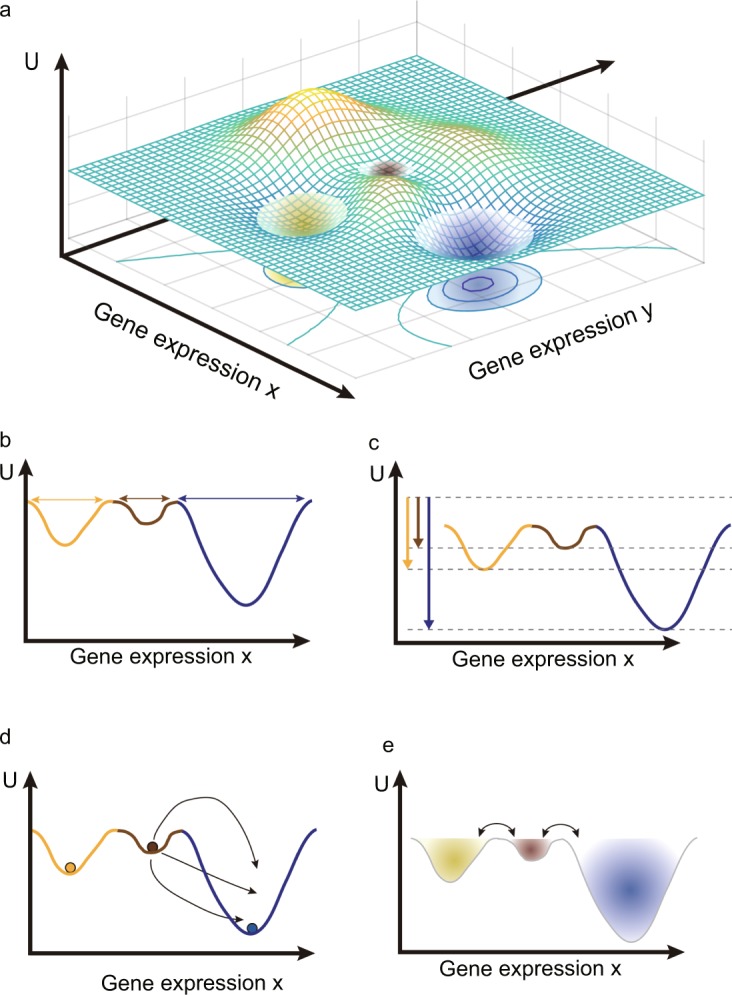
The scheme of quasi-potential landscape and relative dynamic stability. (**a**) A three-dimensional quasi-potential landscape. A point of XY plane is corresponding to a gene expression state of cell and elevations of each point *U*(*x*, *y*) represent the quasi-potential. Three attractors are local minimums in the quasi-potential landscape. (**b**–**e**) are schematic descriptions of four different methods to calculate the relative dynamic stability of the attractors. For clarity, we used two-dimensional representation of the quasi-potential landscape. (**b**) The relative dynamic stability is defined by the basin size. (**c**) The dynamic stability is defined by the steady state probability distribution function. (**d**) The dynamic stability is defined by the mean first passage time. (**e**) The relative dynamic stability is defined by the basin transition rate.

The GRN of a genome produces a multitude of attractor states that have long been proposed to explain the discreteness and robustness of all the cell types of a metazoan organism^19^. The developmental relationship between these attractors is often depicted using Waddington’s pictorial epigenetic landscape, in which attractor states would correspond to the valleys^20^. The epigenetic landscape, however, is more than a metaphor because a GRN’s dynamics can indeed be represented as a quasi-potential function *U*(***x***) that can be theoretically constructed from the systems equations describing the GRN’s dynamics, $\dot{{\boldsymbol{x}}}={\boldsymbol{F}}({\boldsymbol{x}})+{\boldsymbol{\eta }}({\boldsymbol{t}})$, which ***η***(***t***) represents unbiased noise function (Fig. 1a)^21–28^. In the quasi-potential landscape, each point represents a gene expression state ${\boldsymbol{x}}=({x}_{1},\,{x}_{2},\cdots ,{x}_{n})$ and the associated quasi-potential function *U*(***x***); *U*(***x***) informs about the system’s ability to undergo non-equilibrium state transitions even if $\dot{{\boldsymbol{x}}}={\boldsymbol{F}}({\boldsymbol{x}})+{\boldsymbol{\eta }}({\boldsymbol{t}})$ is not a gradient system. In brief, from the Freidlin-Wentzell large deviation theory based on stochastic system^29^, the “relative dynamic stability” can be defined by comparing probability of transitions between attractors which involve a noise-driven exit from an attractor to initiate a move into a neighboring attractor. Mathematically, when we take the limit of noise to zero, it has been shown that *U*(***x***) of a deterministic system $\dot{{\boldsymbol{x}}}={\boldsymbol{F}}({\boldsymbol{x}})$ is related to transition probability of Freidlin-Wentzell stochastic system and represents the least action needed to work against the regulatory constraints imposed by the GRN (which produce the attractor states in the first place)^21^. This theoretical approach assumes Markov property and ergodicity, but the real biological system may not exactly satisfy Markov and ergodic assumptions, for instances, DNA methylation leads to memory effects (non-Markovian) and cells may not visit all possible gene expression states (nonergodic). However, the dynamical system $\dot{{\boldsymbol{x}}}={\boldsymbol{F}}({\boldsymbol{x}})$ is a first order approximation to explain the fundamental principle why the same genome can produce multicellular organism.

Since the construction of continuous models of biological system using $\dot{{\boldsymbol{x}}}={\boldsymbol{F}}({\boldsymbol{x}})$ requires the specific functional form of *F*_*i*_ for each gene *i* in the GRN and associated parameters, which are typically unknown, discrete Boolean network models are frequently used to model GRNs^30–33^. Experimental work and known regulatory modalities can suggest an appropriate Boolean function in lieu of *F*_*i*_, hence facilitating model construction^34–36^. The problem is that a quasi-potential landscape *U*(***x***) cannot be easily defined in the discrete model. Instead of the quasi-potential *U*(***x***), several quantities have been developed to define the relative dynamic stability of a cell state in discrete Boolean network models^30,31,33,37^. In the simplest formalism, the basin size of an attractor serves as an approximation of the relative dynamic stability of a given attractor, that is, the number of discrete states converging to that attractor (Fig. 1b)^33^. Even though the basin size is defined in deterministic condition, a large basin may imply that perturbed states of an attractor x* by noise (e.g. “bit flip” of the value of a gene *x*_*i*_ from 0 to 1 or vice versa) would remain in the basin of the attractor. However, basin size is an inadequate measure for relative attractor stability since attractors with the equal size basin, depending on many other properties, may exhibit distinct resilience to random perturbations^34,38,39^.

As a more appropriate relative stability index, the steady-state probability distribution function (SSP) has been proposed as an estimate of a quasi-potential in discrete Boolean network models^37^. SSP is the probability density function *P*(***x***) for the probability that in a stochastic system, at equilibrium (the steady state) a cell is observed to stay around the attractor. An inverse function of *P*(***x***) can then be visualized as the depth of an attractor basin in a 3D landscape (Fig. 1c), However, the resulting landscape reflects steady-state properties; to establish a stability measure that is not limited to behaviors at equilibrium but quantifies the probability of exit from the attractor, the mean first passage time (MFPT) has been proposed to be used in a non-equilibrium state; MFPT is the average time required for a cell to exit from an attractor state so as to reach another attractor state, which considers all trajectories of cell state transitions that could be used in this process (Fig. 1d)^31^. From the MFPT, the stability difference (relative stability) and thus the preferred transition direction between two attractor states can be inferred. To generalize the relative stability from an attractor to an attracting basin, we can consider transitions between not only attractor states but also any “non-attractor” states (e.g. on transients) which may be located in separate attracting basins (Fig. 1e)^30^. We defined the probability for such a generalized transition as basin transition rate (BTR). Although these various measures for dynamic stability had been developed for the Boolean model, to our best knowledge, these relative stability measures have not been systematically compared for a concrete biological question and have been rarely applied in the large-scale Boolean models due to their computational complexities.

In this paper, we constructed a simple discrete Boolean network model for the epithelial (E)-to-mesenchymal (M) transition (EMT), a biologically important cell state transition that is generic (not tissue specific) and plays a role in development, wound healing and regeneration of various processes and also has been implicated in cancer progression^40^. Recently mathematical models using ODEs to model the underlying gene regulatory network that governs this circuit as well as experimental observations suggest that EMT is not simple binary switching between two stable states E and M, but may involve metastable intermediate states, referred to as hybrid states^41–44^. The relative dynamic stability of three cell states (Epithelial, Mesenchymal and Epithelial/Mesenchymal Hybrid cells) can be generated by the GRN which also determines the transition probabilities. Using a Boolean network to model EMT, we systematically compared the above four measurements of relative dynamic stability (basin size, SSP, MFPT and BTR) and difference between them as well as the reasons for these differences. From that, we propose a new stability index for relative dynamic stability of attractor state. Our results could be useful in studies of cell state transitions, helping to choose the appropriate state stability quantity that is best suited for particular biological questions.

## Results

### Boolean network model for EMT

To compare the different measures for relative dynamic stability, we reconstructed a Boolean network model for a gene regulatory circuit that controls EMT and used the methods above to analyze the relative dynamic stability of various cell attractors in the model. EMT is a process by which epithelial cells lose their cell polarity and cell-cell adhesion and gain migration and invasion capabilities to become mesenchymal cells. This process has been implicated in tumor metastasis, the major cause of death of cancer. More recently, in studies of EMT in breast cancer, a third distinct cell state has been observed in addition to the epithelial (E) cell state, and the mesenchymal cell (M) state: an intermediate “hybrid” (H) cell state which displays both epithelial and mesenchymal features with regard to gene expression patterns^41,42^. The existence of this additional state with promiscuous gene expression pattern has been postulated independently based on dynamical gene circuit models^43,44^, and it is likely that additional distinct states are produced by the multi-stable dynamics of the underlying gene regulatory network. The three-state model provides an ideal benchmark system for our comparison of the four measures of dynamic stability because having more than two stable states offers a richer opportunity to order the attractors according to dynamic stability of and to examine its biological significance.

**Figure 2:**
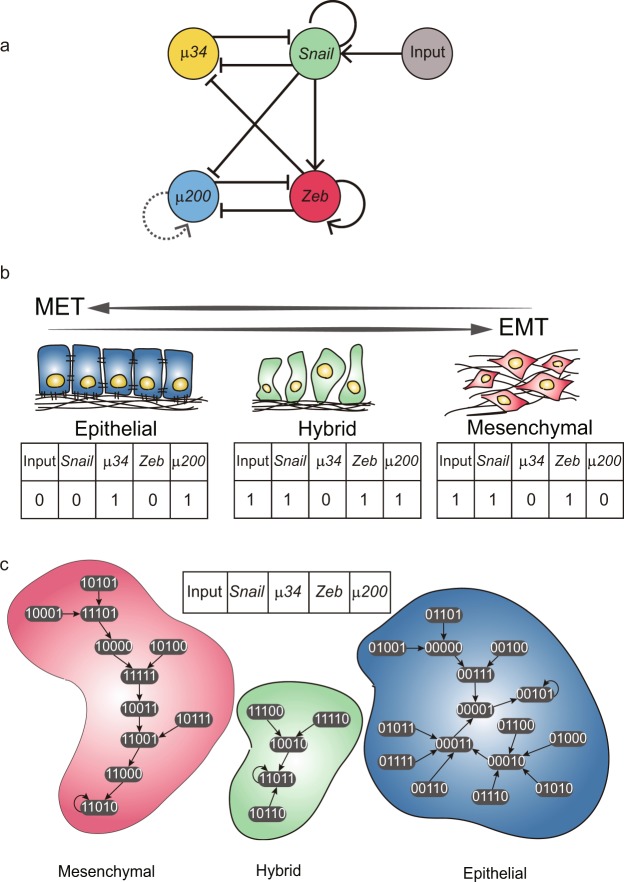
The EMT Boolean network model and three cell attractors and attracting basins. (**a**) A network structure for the EMT model. The sharp arrows represent the activating regulations while the blunt arrows represent the repressing regulations between nodes. A dashed link is for a proposed regulation in this model. (**b**) The definitions of three cell attractors in Boolean state. (**c**) Transition map of the EMT Boolean model. Each ellipse represents one Boolean state of the model. Without internal and external perturbation, state changes of the EMT model follow arrows due to the Boolean functions. There are three cell attractors and each basin of them is distinguished by different colors: blue for epithelial cells, red for mesenchymal cells and green for hybrid cells.

Establishing a Boolean network model requires that one constrains the gene regulatory interactions (network topology) and the gene expression profiles of each attractor to the experimental observations. The third piece of information is also needed: the Boolean logic function controlling expression of each gene is currently not readily observed and must be inferred (see below). We first surveyed the literature^43^ and identified four key genes regulating EMT: *miR-*34 (*μ34*), *miR-*200 (*μ200*), *Snail* and *Zeb* (Fig. 2a). They form two mutually inhibitory toggle switches (*μ*34 vs. *Snail* and *μ*200 vs. *Zeb*). *Snail* and *Zeb* form a coherent feed-forward loop and a positive feed-back loop involving *μ*200 and *μ*34, respectively. One input node is added to four gene circuit to represent the external signals which induce EMT, thus obtaining a *n* = 5 nodes GRN. Before defining the Boolean logic function of each node, we first defined the Boolean state x* of the three attractors. *μ*200 is an epithelial marker whereas *Zeb* is a mesenchymal marker, and we assumed that a cell in the hybrid state would express both markers of epithelial and mesenchymal cells. Thus, we defined the epithelial state, hybrid state and mesenchymal state as (Input, *Snail*, *μ*34, *Zeb*, *μ*200) = (0, 0, 1, 0, 1), (1, 1, 0, 1, 1) and (1, 1, 0, 1, 0) respectively (Fig. 2b).

We tried to identify Boolean functions of each gene based on the experimental observation and known biological mechanism of genes, but they failed in producing the defined three attractors. Hence, we generated all possible Boolean functions to identify the combination of Boolean functions associated with all the five nodes which produces the three defined attractors as point attractor states. As there are a large number of possible Boolean functions, to reduce the search space of Boolean functions, we used only a subset of Boolean function called nested canalizing Boolean functions (NCBFs, see Methods for details). The outputs of NCBFs can be determined by subsets of input arguments and so they exhibit stable dynamics which as usually observed in biological systems^31,45–47^. Moreover, we added the proposed self-regulating link to the node *μ*200 (Fig. 2a) which helped the mutual inhibitory circuit of *Zeb* and *μ*200 to generate three stable attractor states^43^. We were able to identify three Boolean models consisting of NCBFs which produced the three defined point attractors. However, two models included wrong NCBFs that *μ*34 activated *Snail* and *μ*200 could not inhibit *Zeb*, respectively, which were different from the known role of *μ*34 and *μ*200. Thus, we used the remained set of NCBFs to EMT Boolean model which agreed well with the known biology of genes (See details of the logic tables in Supplementary Table S1). Including the input node which sustains its initial value, our EMT Boolean model has 32 (2^5^) states. These 32 states converge to three attractors which we defined as the epithelial, mesenchymal and hybrid attractor states, dividing the state space into three basins of attraction (Fig. 2c).

### Validation of EMT Boolean network model with knock-out and overexpression experiments

Besides showing that the EMT Boolean model is valid in that it correctly represents the three stable cell states, we also validated our model against results from gene knock-out (KO) or over-expression (OE) experiments. We simulated the genetic mutations by setting the Boolean function of the manipulated node (mutated gene) in the network to return 0 (KO) or 1 (OE), irrespective of values of the other nodes. The genetic mutations caused new attractors to appear - we defined these novel attractor states using the values of *μ*200 and *Zeb*: an epithelial-like attractor (EL, *μ*200 = 1, *Zeb* = 0), a mesenchymal-like attractor (ML, *μ*200 = 0, *Zeb* = 1), a hybrid-like attractor (HL, *μ*200 = 1, *Zeb* = 1), and an undefined attractor (ND, *μ*200 = 0, *Zeb* = 0).

Simulation results were compared with experimental observations (Table 1). Increased expression of *MIR*200 family transcripts has been reported to induce mesenchymal-to-epithelial transition (MET, the reverse of EMT), and to suppress EMT, invade, and metastasize^48,49^. Transfection of mesenchymal cells with *MIR*200 family caused morphological change from mesenchymal to epithelial-like form with many cells aggregating together in groups^50^. Our EMT Boolean network recapitulated this observation: the simulations showed that the mesenchymal attractor state disappeared if we set the value of the *μ200* node to 1. On the other hand, anti-*MIR200* treatment resulted in increased metastases *in vivo*, and caused an increase in the migration of cells and mesenchymal characteristics *in vitro*^48,50^. This finding was reproduced in the simulation where setting to the *μ200* node value 0 resulted in a state space that had only mesenchymal and mesenchymal-like attractors. Similarly, when *MIR*34*a* was ectopically introduced into cells, *Snail1* protein levels and E-cadherin promoter activity decreased to baseline and mobility of the cell was decreased^51^; our model reproduced this observation because an epithelial point attractor and a cyclic attractor with repeating non-mesenchymal state was generated by setting the *MIR*34 node to the value 1. Conversely, re-expression of *SNAI1* resulted in the motile response of *MIR*34*a*-expressing cell^51^ which agrees with our corresponding simulation of up-regulating *Snail*: the resulting state space contained mesenchymal and hybrid attractors but no epithelial attractor. Lastly, simulation of over-expression of *Zeb* resulted in the removal of the epithelial attractor, which is consistent with the observed association of the induction of EMT in breast cancer cells with increased expression of *ZEB1/ZEB2*^48^. These comparisons with experimental findings demonstrated that our EMT Boolean network model not only correctly generated the three cell attractor states that produce biological characteristic gene expression patterns but also recapitulated the effects of genetic mutations of the gene regulatory network that governs EMT.

**Table 1:** Cross validation of EMT model from knock-out or over-expression experiments.

Node	UP/DOWN	Experiment	Simulation results		Ref.
			Epithelial	Mesenchymal	
*μ*200	UP	Reversal of EMT	Yes	No	^48^
*μ*200	DOWN	EMT	No	Yes	^48^
*Zeb*	UP	Induction of EMT	No	Yes	^48^
*μ*200	UP	Abrogate EMT	Yes	No	^49^
*μ*200	UP	MET	Yes	No	^50^
*μ*200	DOWN	EMT	No	Yes	^50^
*μ*34	UP	MET	Yes	No	^51^
*Snail*	UP	EMT	No	Yes	^51^

KO and OE simulation results of EMT Boolean model are validated by experiment results. Simulation results column shows existence of epithelial attractor and mesenchymal attractor.

### Computing the relative dynamic stability of the three cell attractors in EMT using the four known methods

Using the validated EMT Boolean model, we calculated the relative dynamic stability of cell attractors based on the four methods described above: (*i*) the attractor basin size, (*ii*) the steady state probability distribution function (SSP), (*iii*) the mean first passage time (MFPT) and (*iv*) basin transition rate matrix (BTR). The purpose is to compare the different methods to measure the stability of the attractors, i.e. their ability to determine the relative stability. For calculating SSP, MFPT and BTR, we constructed a stochastic model, which is identical to the deterministic EMT Boolean model biologically, by introducing biological gene expression noise *η* = 0.01 to the deterministic model (for details, see State transition matrix and steady state probability density function in Methods). The relative attractor stability has a functional (measurable) consequence in that it affects the preferred directionality of attractor transitions (EMT, MET) and hence can be benchmarked against experiments. This will allow us to determine which of these four methods best matches experimental results, which in turn will help to design better measures for the relative dynamic stability.

**Figure 3:**
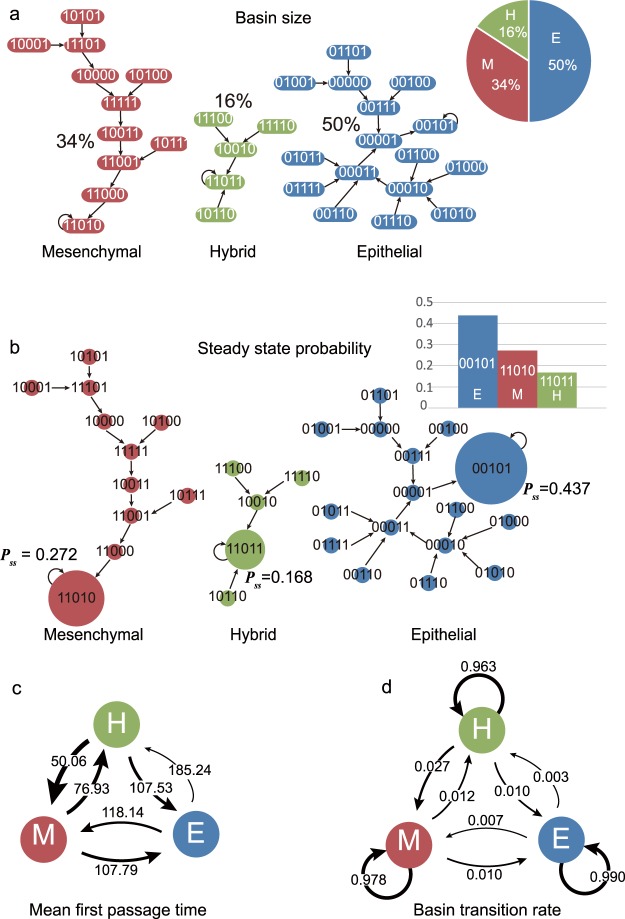
The relative dynamic stability of the attractors in the EMT Boolean model computed by four different methods. (**a**) The basin size of three attracting basins. The basin size is a count of states (ellipses) having the same color with a cell attracting basin. Basin size of each attractor is represented by a ratio to the number of all possible states. (**b**) Steady state probability distribution functions *P*_*ss*_ of three cell attractor states. The size of circle represents the steady state probability of each state. Non-attractor states have very small probabilities. (**c**) Mean first passage time between three cell attractor states. Width of an arrow is thicker as MFPT is shorter because the shorter is MFPT, the easier is the transition. (**d**) Basin transition rates between three cell attractor states. Contrary to MFPT, width of each arrow is thicker as BTR is higher.

The attractor of the epithelial cell state has 16 network states (50%) in its basin (Fig. 3a). The mesenchymal attractor has a basin of 11 states (34%) whereas the hybrid attractor has a basin of only 5 states (16%). Using the attracting basin size as a measure, we can rank the attractors for their relative dynamic stability: the epithelial cell is the most stable one. For the relative dynamic stability calculated using SSP, only the attractor states have meaningful values whereas other states have almost zero values. The epithelial, mesenchymal, and hybrid attractors have the steady-state probabilities *P*_*ss*_ as 0.437, 0.272, and 0.168 respectively (Fig. 3b). The steady state probability distribution *P*_*ss*_ shows that the epithelial attractor is the most stable state whereas the hybrid attractor is the least stable one. This is consistent with the experimental characterization of the hybrid cell state as a metastable and stem-cell like intermediate state^42^.

Rather than measuring the relative dynamic stability using a quantity for each attractor separately, such as basin size and steady state probability, the other two methods, MFPT and BTR, consider pairwise relationships to define the stability. As shown in Fig. 3c, the MFPT values for the transitions between epithelial and hybrid attractors are, for the two directions, 182.53 (Epithelial to Hybrid) and 107.53 (Hybrid to Epithelial). Thus, the epithelial attractor is more stable (since cells stays on average longer in it) than the hybrid one. Similarly, since the MFPT from the mesenchymal attractor to the hybrid attractor is larger than the one of the backward transition, 76.93 and 50.06, respectively – the mesenchymal attractor is more stable than the hybrid attractor. Lastly, the epithelial attractor is more stable than the mesenchymal attractor because the transition from the epithelial attractor to the mesenchymal attractor takes longer than the transition in the opposite direction (118.14 and 107.79, respectively). Importantly, this triangle of net preferred direction is transitive, allowing us to rank the three attractors: The epithelial cell is the most stable while the hybrid cell is the least stable based on MFPT, which agrees with the previous measurements.

Finally, we examined the basin transition rates. From the basin transition matrix for the BTR values, we can compare two properties of basin transition: self-transition rate and relative transition rate (Fig. 3d). The epithelial, mesenchymal, and hybrid basin have the self-transition rates of 0.990, 0.978, and 0.963 respectively. Between the epithelial basin and mesenchymal basin, the transition from the epithelial basin to the mesenchymal basin is the preferred one. The transition from the epithelial basin to the hybrid basin is also preferred to the backward transition. Lastly, transition from the mesenchymal basin to the hybrid basin is less probable than the transition from the hybrid basin to the mesenchymal basin. To compare the relative stability associated with each basin, we need to consider the relative transition rates. This allowed us to compare the relative stability: The epithelial basin is the most stable while hybrid basin is the least stable among three basins (Fig. 3d).

In conclusion, all four methods for calculating the relative dynamic stability were in agreement with each other in ranking the three cell attractors according to their relative stability. These results are supported by experimental observations: the hybrid cell state has stem cell-like property and tends to spontaneously differentiates to epithelial and mesenchymal cell states, suggesting that it is the least stable^42,52^. Mesenchymal cells tend to transform into the epithelial state spontaneously - which supports the simulation result that the epithelial cell is the most stable cell type^53^.

### A new stability index based on 1-degree neighborhood graph

Next, we compared the four measurements of relative dynamic stability for the cell attractors in the EMT Boolean network model with respect to genetic mutations. We knocked-out or overexpressed each gene and measured again the relative dynamic stability. Interestingly, differences appeared between the four methods (Supplementary Figs S1 and S2 and Supplementary Table S2). For instance, in the *μ34* knock-out (KO) simulation, the hybrid and the mesenchymal cell states were equivalently stable if basin size and BTR were used as a measure of relative stability, whereas the mesenchymal cell state was more stable than the hybrid cell state if SSP and MFPT were used (Supplementary Fig. S1c,h,l and p).

**Figure 4:**
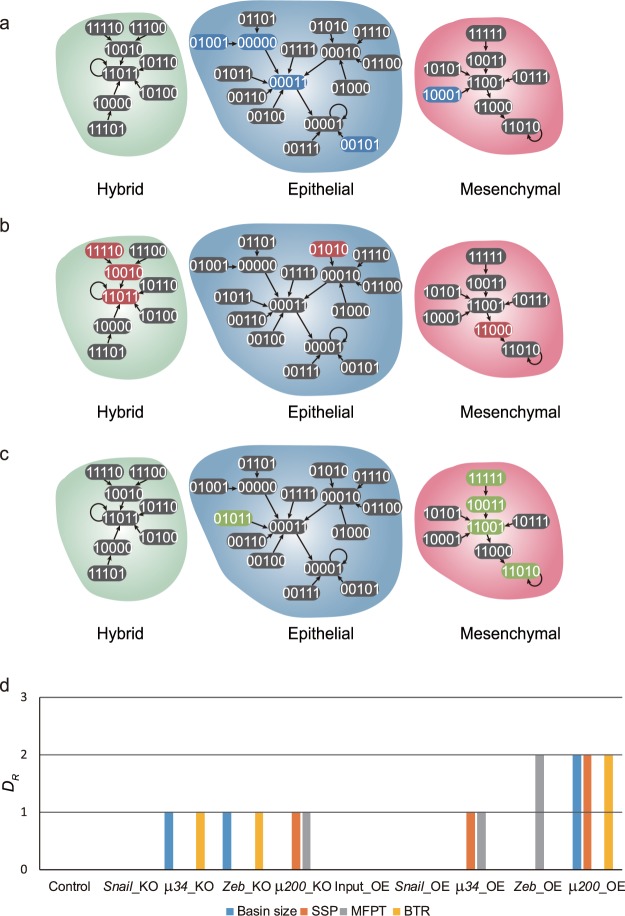
The 1-degree neighbor distribution and the stability index for EMT Boolean network models with various mutations. (**a–c**) The 1-degree neighbor distribution graphs for three cell attractors of the *μ*34 KO model. 1-degree neighbors of each attractor *A* state are highlighted with the same color as the respective attractors *A*. (**a**) 1-degree neighbors of the epithelial cell attractor state (blue), (**b**) 1-degree neighbors of the mesenchymal cell attractor state (red), (**c**) 1-degree neighbors of the hybrid cell (green). (**d**) Differences between the stability index *S*_*A*_ and other measures of relative dynamic stability. The value 0 of *D*_*R*_ indicates that the stability index produces the same result with other measure *R*.

To understand the cause of this discrepancy, we considered *one-degree neighbor states* (1-degree neighbor) as shown in Fig. 4a–c. A 1-degree neighbor of a given attractor state is the state whose n-bit state vector has one-bit value difference (Hamming distance of 1) from that of the attractor state. The 1-degree neighbor may reside in the same basin or in another basin of attraction. Because transitions from an attractor state to its 1-degree neighbor are the most probable transitions (except the transition to itself), the distribution of these 1-degree neighbors of each attractor in the various basins can predict the major trajectories of attractor transitions driven by random noise. When we use basin size as a measure of relative dynamic stability, we usually think that the perturbed states from an attractor are uniformly distributed and a larger basin would contain more the perturbed states. However, we found that the distribution of 1-degree neighbors of each attractor was independent of its basin size. Moreover, the relative dynamic stability analysis using BTR on the *μ34* KO model did not agree with our transition trajectory analysis based on the 1-degree neighbors. For instance, BTRs between mesenchymal and hybrid states were equivalent, however, the distribution of 1-degree neighbors of these two states were not same; four 1-degree neighbors of the hybrid state were in the mesenchymal basin (Fig. 4c) whereas only three 1-degree neighbors of mesenchymal state were in the hybrid basin (Fig. 4b). Interestingly, MFPTs between mesenchymal and hybrid states agreed with the biased distribution of 1-degree neighbors of two cell states. The relationship between SSP and 1-degree neighbor distribution is more complex than other measurements because SSP is the result of the equilibrium state at longer time scale whereas the 1-degree neighbor distribution only predicts the results of the dynamics in the short time scale. However, we can infer that SSP of an attractor is high when many 1-degree neighbors of all the attractors locate in the basin of that respective attractor.

From this, we propose a new stability index *S*_*A*_ for relative dynamic stability of an attractor *A* in the Boolean network model as follows:

1 $${S}_{A}=\sum _{i}{O}_{i}^{A}-\sum _{j\ne A}{O}_{A}^{j}$$

where ${O}_{i}^{j}$ is defined as a ratio of 1-degree neighbors of attractor *i* in the basin of attractor *j* to total 1-degree neighbors of attractor *i* (Supplementary Table S3). The larger the stability index *S*_*A*_, the more stable is an attractor state in a Boolean network model. To verify the relation between the stability index and relative dynamic stability, we compared the new stability index with other stability measurements (Fig. 4d and see Material and Method for details). In the cases of *μ34* knock-out and *Zeb* knock-out networks, basin size and BTR produced different rankings of attractor stability than by SSP and MFPT (Supplementary Figs S1 and S2). By contrast, the stability index *S*_*A*_ reproduced the rankings obtained by using SSP and MFPT (Fig. 4d). However, in the cases of *μ*200 knock-out and *μ*34 over-expression networks, the stability index *S*_*A*_ failed to produce the same stability ranking as SSP and MFPT. Since the differences of attractors in the *μ*200 and *μ*34 mutated networks were very small (Supplementary Figs S1j,n and S2h,m), we propose that the biased distribution graph of 2-degree neighbors or higher-degree neighbors will explain these slightly different results. In the *Zeb* over-expression network, using MFPT resulted in ranking of attractor stability that was different from other measurements (Fig. 4d). In the *μ*200 over-expression network, our stability index *S*_*A*_ agreed with MFPT but differs from the methods based on basin size, SSP and BTR. Overall, our simple stability index agreed with the most of other methods to calculate the relative dynamic stability of Boolean models of various mutated networks.

**Figure 5:**
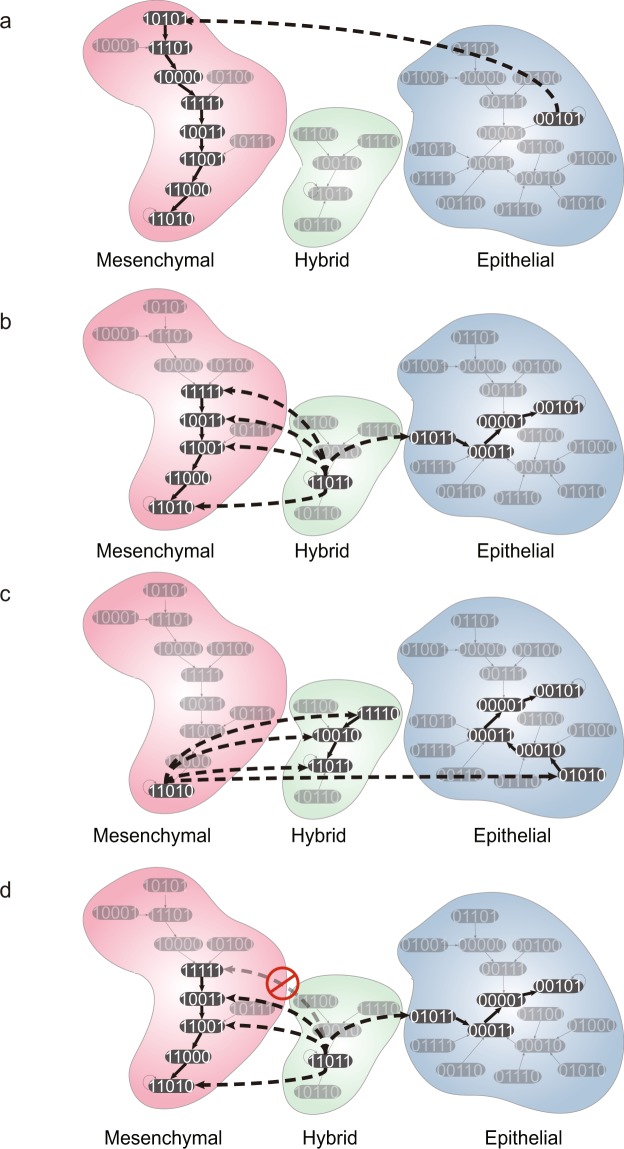
The Analysis of major transition trajectories between the three cell attractors of EMT Boolean model. The major transition trajectories from (**a**) epithelial cells, (**b**) hybrid cells and (**c**) mesenchymal cells to other cell state attractors. The dashed bold arrows represent the escaping transition driven by noise and the solid bold arrows represent the following sequential transitions due to execution of Boolean functions. (**d**) This example demonstrates that inhibition of one single node *µ34* (third position in the state vector) is not sufficient to block the transition from hybrid to mesenchymal attractor.

From the 1-degree neighbor graph, we can identify the major trajectories of transitions between cell states. A major trajectory consists of two stages: (*i*) an escaping transition from a starting attractor to one of its 1-neighbor in the basin of end attractor due to noisy fluctuation of a gene expression, and (*ii*) following sequential transitions toward the end attractor actuated by Boolean functions. The epithelial cell state had one major trajectory toward the mesenchymal state but no major trajectory toward the hybrid state (Fig. 5a). Both mesenchymal and hybrid states had one major trajectory toward epithelial state (Fig. 5b and d); the mesenchymal state had three major trajectories to the hybrid state and the hybrid state could transit to mesenchymal state along four major trajectories (Fig. 5b and c). Especially, one of the trajectories from the hybrid to mesenchymal state started by the activation of *μ*34 (third position in the state vector). If we prevented the activation of *μ*34 in hybrid cell state, it would block one major trajectory, but the other trajectories would remain, so the differentiation of hybrid state would not be blocked (Fig. 5d). This observation may confirm the widely held notion that manipulation of gene expression of a single gene (node) to achieve a cell attractor state transition is not sufficient^3^.

## Discussion

In this work, we constructed a Boolean network model for the EMT process and analyzed the relative dynamic stability of attractor states using various quantities. Four relative dynamic stability measures found in the literature were applied to examine our EMT Boolean network model. They agreed with each other in the wild-type networks but displayed different results in the cases with the genetic mutations that permanently altered the activity of individual genes. From this observation, we propose a new index for the relative dynamic stability of Boolean network by analyzing the graph of 1-degree neighbors of the attractors.

The four stability measures are based on different assumptions which naturally can lead to different results. First, basin size assumes fully deterministic dynamics whereas the other measures assume stochastic dynamics. Second, basin size and BTR assume that a basin represents a biological cell state but SSP and MFPT assumes that the cell state is only the attractor state no matter whether it is a limit cycle or a fixed point attractor. In the case of limit cycles, we used the sum of SSP of each state in a cyclic attractor to compare with SSP of other attractor, but it is still required to be further studied whether SSP of each state in cyclic attractor or the sum of them is meaningful. Finally, SSP assumes that the probability at the attractor state is the most important whereas MFPT assumes that relative difference of attractor transitions is the most significant. These assumptions contribute to the differences in the simulation results of mutations of a Boolean network. Hence, people should choose the appropriate method among four methods according to the purpose and model of the analysis.

When we consider a large-scale Boolean network model, there are additional problems with the currently available methods to calculate relative dynamic stability of attractors. Because computational complexity increases exponentially with the size of the network, it will be difficult to enumerate all the states to determine the basin size, and to calculate steady state probability function, mean first passage time or the transition rates between attractor states. However, our proposed stability index is simple to calculate which enable us to apply it to large-scale Boolean network models once the attractor states are identified. In large-scale networks, the noise level *η* is important to the stability index. A probability of transition to 1-degree neighbors is *N* × *η*(1 − *η*)^*N*−1^ but the transition probability to 2-degree neighbors is $\frac{N(N-1)}{2}\times {\eta }^{2}{(1-\eta )}^{N-2}$ for a network of size *N*. Therefore, when *ηN* ≪ 1 – *η*, transition probability to 2-degree neighbors is much lower than the transition probability to 1-degree neighbors, and our dynamic stability index defined by only 1-degree neighbors can be reliable. In other words, when the dynamic stability indexes are different among attractors, 2-degree neighbors can be ignored. However, when the stability indexes of two attractors are identical, 2-degree neighbors can make a slight difference of the relative stability of them (Fig. 4d).

We added an auto-regulation link on *μ*200 even though there is no identified auto-regulation of *μ*200 (Fig. 2a). However, the added auto-regulation is matched to the largest parameter of synthesis rate of *μ*200 in ODE model^43^. Interestingly, the added auto-regulation cannot cancel inhibitory effect of *Zeb* and *Snail* when one of them is activated and another is inactivated, but the auto-regulation of *μ*200 can buffer the inhibitory effect of *Zeb* and *Snail* when both are activated (Table 1). This is not general but seems to be required to construct tertiary stable toggle switch in Boolean model.

We used the synchronous updating Boolean model in this research. However, there are many researches using asynchronous updating Boolean models or multi-value logical model^54–59^. These researchers have identified attractor states but not analyzed relative stability of attractors due to lack of stability measure in their models^60^. Even though intrinsic and extrinsic noise is not defined in asynchronous model generally, 1-degree neighbors can be defined as in synchronous model and hence the suggested stability index may be relevant in asynchronous model. Moreover, if we define 1-degree neighbors considering multi-value of node, the stability index can be applied in multi-value logical model as well.

Genetic mutations and epigenetic modifications modify the Boolean functions and hence the landscape of gene regulatory network. However, as in Fig. 5, transient perturbations cannot modify the Boolean function and the landscape, but they can repress or promote some trajectories of cell state transition; it is possible that undesired cell state transition can occur through unaffected trajectories. Moreover, if the desired cell state is less stable than other cell states, then undesired cell state transition will occur by intrinsic and extrinsic noise.

In Boolean network model, the stable motifs associated to an attractor should be controlled to reach the attractor from any initial states^58^. Among 10 cases with a single mutation, only one case (Input_KO) resulted in a single attractor of state space (Figs S1 and S2). From that, we can predict that most of induced cell state transitions may result in multiple attractors including undesired ones and hence the efficiencies of them is usually low. To improve the efficiency, we need to make the desired attractor as a unique attractor of the state space^61^ or as the most stable attractor relatively. Therefore, computing the relative dynamic stability in Boolean networks model can be relevant in the study of the cell state transition, such as cell differentiation, cell reprogramming, cancer metastasis and drug resistance.

## Methods

### Boolean network model and attracting basin size

The Boolean network model, among the historically oldest discrete model for dynamical systems, was firstly proposed by S. A. Kauffman^19^. Boolean networks represent the dynamics of an influence (regulatory) network in which the nodes influence the activity values of each other via the edges. Nodes can take the state being off or on, taking the Boolean values (0 or 1); edges represent the regulatory interactions (influences) between two nodes and represent the modality of the influence (e.g. activation or inhibition). The latter is formalized by the Boolean function *f*_*i*_ associated with each node *i* which integrates the values of its input nodes to update its value. Thus, to represent the states of *n* nodes of a Boolean network, a state vector ***x***(*t*) at specific time *t* is defined as: ${\boldsymbol{x}}(t)\,=\,({x}_{1}(t),\ldots {x}_{i}(t),\ldots ,{x}_{n}(t))$, *n* is the number of nodes of a network. Because the value of node is either 0 or 1, ***x***(*t*) is represented as a *n*-bit binary vector. The value of node *i* is updated at each discrete time step by a Boolean function ${f}_{i}({{\boldsymbol{x}}}_{j\in {I}_{i}})$; *I*_*i*_ is the set of upstream regulators that serve as input of node *i*, ${{\boldsymbol{x}}}_{j\in {I}_{i}}$ is a state vector of all regulating nodes. Using the Boolean functions, we can calculate a state vector ***x***(*t* + 1) which is the unique state of ***x***(*t*):

2 $${\boldsymbol{x}}(t+1)={\boldsymbol{F}}({\boldsymbol{x}}(t))=({f}_{1}({{\boldsymbol{x}}}_{j\in {I}_{1}}),\ldots ,{f}_{n}({{\boldsymbol{x}}}_{j\in {I}_{n}})),$$

***F*** is a state transition function of the entire Boolean network. Note that here values of each node are updated simultaneously, i.e. we employ the synchronous updating policy. Because each node can have two possible values, the state space contains 2^*n*^ possible vector states.

When an initial state ***x***(0) of the network reaches a steady state ***x***(*t*), i.e. ***x***(*t*) = ***x***(*t* + *L*), if *L* = 1, we call the state ***x***(*t*) as a point attractor; if *L* > 1, the states ***x***(*t*), ***x***(*t* + 1), …, ***x***(*t* + *L* − 1) represents a limit cycle. A Boolean network can have multiple point attractors and limit cycles - every point in the state space converges to one of them. The basin of an attractor is the set of states ***x***(0) that will converge to the same attractor. Therefore, the basin size of an attractor is the number of states in the basin of an attractor.

### State transition matrix and steady state probability density function

Boolean networks can be used to model stochastic gene expression noise^62^, in which, according to some scheme the values of each node randomly (spontaneously) flip to the alternative state. In such models the network can undergo spontaneous transitions stochastically between two attractors due to the intrinsic noise in the system. Since the transition probability between any two states can be calculated from the driving force ***F***(***x***(*t*)), we can use a discrete Markov model to trace the stochastic transitions of the whole system at any time. The transition matrix *T* in the Markov model can be obtained from the Boolean functions ***F***(***x***(*t*)) in a deterministic manner: an entry *T*_*lm*_ is 1 only if a state *l* is the successor state of a state *m* according to ***F***(***x***(*t*)), otherwise, *T*_*lm*_ = 0. Since gene expression dynamics is stochastic, with this implementation it is possible that state transitions sometimes do not follow ***F***(***x***(*t*)). To represent this stochastic process, let *η* to be a probability in which noise changes one bit in the *n*-bit binary vector, then the stochastic transition matrix *P*_*lm*_ according to noise is as follows^63^:

3 $${P}_{lm}=\{\begin{array}{c}{C}_{H(l,m)}^{n}\,{\eta }^{H(l,m)}\cdot {(1-\eta )}^{n-H(l,m)}(l\ne m)\\ 0\,(l=m)\end{array}$$

*H*(*l*, *m*) is the Hamming distance between two states *l* and *m*. The stochastic transition matrix ${T}_{lm}^{\ast }$ is defined as follows:

4 $${T}_{lm}^{\ast }={(1-\eta )}^{n}\cdot {T}_{lm}+{P}_{lm}$$

Then T* contains the state transition probability between network states which is driven by both the deterministic force ***F***(***x***(*t*)) and the noise *η*. We define a 2^*n*^-dimensional vector ***P***(*t*) as the probability distribution function of all states in the network at specific time $t$, and we can get ***P***(*t* + 1) at the next time step by using T*:

5 $${\boldsymbol{P}}(t+1)={{\boldsymbol{T}}}^{\ast }\cdot {\boldsymbol{P}}(t)$$

After long enough time, the probability distribution function will reach a steady state ***P***_*ss*_:

6 $${\boldsymbol{P}}(t+1)={\boldsymbol{P}}(t)={{\boldsymbol{P}}}_{ss}$$

where ***P***_*ss*_ is called the steady state probability distribution function of a Boolean network.

### Mean first passage time (MFPT)

The Mean First Passage Time (MFPT) *M*_*ij*_ is the expected time steps for the occurrence of the first passage from a state *j* to another state *i* in a stochastic process^64^. Let ${f}_{ij}^{n}$ be the probability that first passage time from *j* to *i* is *n* time steps. Then, we can define the transition probability ${{\rm{p}}}_{ij}^{n}$ from an initial state *j* to a final state *i* in *n* time steps as follows:

7 $${{\rm{p}}}_{ij}^{n}={f}_{ij}^{n}+{f}_{ij}^{n-1}\times {{\rm{p}}}_{jj}^{1}+{f}_{ij}^{n-2}\times {{\rm{p}}}_{jj}^{2}+\ldots +{f}_{ij}^{1}\times {{\rm{p}}}_{jj}^{n-1}$$

Finally, we have the recursive definition of ${f}_{ij}^{n}$:

8 $${f}_{ij}^{n}={{\rm{p}}}_{ij}^{n}-\sum _{k=1}^{n-1}{f}_{ij}^{n-k}\times {{\rm{p}}}_{jj}^{k}$$

and MFPT *M*_*ij*_ is a sum of time step *n* times its probability ${f}_{ij}^{n}$. By using the fundamental matrix ***Z*** = (***I*** − T* + ***W***)^−1^, where ***I*** is an identity matrix and each column of ***W*** is ***P***_***ss***_, we can get the MFPT matrix:

9 $${{\boldsymbol{M}}}_{ij}=\frac{{{\boldsymbol{Z}}}_{ii}-{{\boldsymbol{Z}}}_{ij}}{{P}_{ss}^{i}}$$

where ${P}_{ss}^{i}$ is the *i*th element of ***P***_*ss*_^65^.

### Basin transition rate matrix

Another method for characterizing the dynamics of a Boolean network is to define a cell type as a basin of attraction and calculate the transition rate between the basins by using Monte Carlo simulation^30^. With this idea, we obtained the basin transition matrix ***B*** by using state transition matrix T*. An entry *B*_*UV*_ is a probability of basin transition from basin *V* to basin *U*:

10 $${B}_{UV}=\sum _{i\in U}\sum _{j\in V}\,{T}_{ij}^{\ast }/size(V).$$

### Nested Canalizing Boolean Function

Since the actual Boolean functions of each node of a Boolean network is difficult to determine, we may need to guess certain structures of them. From the limited biological experiment data, we know that a knock-out or knock-down of a key regulating gene usually decreases the expression of the regulated gene significantly. Thus, we used a subset of Boolean function called nested canalizing Boolean functions (NCBF) which considers this kind of regulatory logic. NCBF satisfy the constrain that there is a permutation of inputs (*σ*_1_, …, *σ*_*n*_) such that:

11 $$\begin{array}{c}f({x}_{{\sigma }_{1}}={a}_{{\sigma }_{1}},\ldots ,{x}_{{\sigma }_{n}})={b}_{{\sigma }_{1}};\\ f({x}_{{\sigma }_{1}}\ne {a}_{{\sigma }_{1}},{x}_{{\sigma }_{2}}={a}_{{\sigma }_{2}}\ldots ,{x}_{{\sigma }_{n}})={b}_{{\sigma }_{2}};\\ \begin{array}{c}\ldots ;\\ f({x}_{{\sigma }_{1}}\ne {a}_{{\sigma }_{1}},{x}_{{\sigma }_{2}}\ne {a}_{{\sigma }_{2}}\ldots ,{x}_{{\sigma }_{n}}={a}_{{\sigma }_{n}})={b}_{{\sigma }_{n}}\end{array}\end{array}$$

where ${b}_{{\sigma }_{i}}$ is the canalyzed Boolean value of input ${x}_{{\sigma }_{i}}$. For example, there is a Boolean function *f*(*x*_1_, *x*_2,_ *x*_3_) = *x*_1_ AND *x*_2_ AND NOT *x*_3_, this is a NCBF because there is a permutation [1, 3, 2] of inputs (1, 2, 3) such that *f*(*x*_1_ = 0, *x*_3_, *x*_2_) = 0; *f*(*x*_1_ ≠ 0, *x*_3_ ≠ 1, *x*_2_) = 0; *f*(*x*_1_ ≠ 0, *x*_3_ ≠ 1, *x*_2_ = 1) = 1 (for this function, there are more permutations of inputs). However, *f*(*x*_1_, *x*_2,_ *x*_3_) = (*x*_1_ AND *x*_2_) OR (NOT *x*_1_ AND *x*_3_) is not a NCBF because there is no such permutation of inputs (1, 2, 3).

### Comparisons between the stability index and other relative stability measures

To compare the stability index with other relative stability measures, we used rankings of attractor states based on the relative dynamic stability:

12 $${D}_{R}=\,\sum _{{A}_{i}}\,{({r}_{S}({A}_{i})-{r}_{R}({A}_{i}))}^{2}$$

where *r*_*S*_(*A*_*i*_) is a ranking of an attractor *A*_*i*_ by using the proposed stability index and *r*_*R*_(*A*_*i*_) is the ranking by another relative stability measure *R*. Hence, *D*_*R*_ shows a difference between the stability index and a relative stability measure *R*.

## Electronic supplementary material

Supplementary Information

Supplementary Table S2

Supplementary Table S3

## Data Availability

All data generated or analysed during this study are included in this published article (and its Supplementary Information files).
