# Supplementary material for: Determining Relative Dynamic Stability of Cell States Using Boolean Network Model
Source: Sci Rep. 2018 Aug 13;8:12077. doi: 10.1038/s41598-018-30544-0 (PMC6089891; doi:10.1038/s41598-018-30544-0)
Supplement: Supplementary file 1 — Supplementary Information [file 41598_2018_30544_MOESM1_ESM.pdf]

Supplementary Information

Determining Relative Dynamic Stability of Cell States Using Boolean Network Model

Jae Il Joo[1§], Joseph X. Zhou[2,3§], Sui Huang[2*], Kwang-Hyun Cho[1*],

[1] Department of Bio and Brain Engineering, Korea Advanced Institute of Science and Technology (KAIST), Daejeon 34141, Republic of Korea
[2] Institute for Systems Biology, Seattle, WA, USA
[3] Kavli Institute for Theoretical Physics, UC Santa Barbara, California, USA

§ These authors contributed equally as first author
*Authors for correspondence (KHC: ckh@kaist.ac.kr; SH: shuang@systemsbiology.org)

Supplementary Table S1. Derived logic tables for Boolean network model for EMT.

Snail(t+1) = Input*(t)* AND NOT (*Snail(t)* AND *μ34(t)*)

**Input(*t*)**	***Snail*(*t*)**	***μ34*(*t*)**	***Snail*(*t*+1)**
0	0	0	**0**
0	0	1	**0**
0	1	0	**0**
0	1	1	**0**
1	0	0	**1**
1	0	1	**1**
1	1	0	**1**
1	1	1	**0**

μ34(t+1) = NOT *Snail(t)* AND NOT *Zeb(t)*

Snail(t)	***Zeb(t)***	***μ34(t+1)***
0	0	**1**
0	1	**0**
1	0	**0**
1	1	**0**

Zeb(t+1) = NOT *μ200(t)* OR (*Snail(t)* AND *Zeb(t)*)

Snail(t)	***Zeb(t)***	***μ200(t)***	***Zeb(t+1)***
0	0	0	**1**
0	0	1	**0**
0	1	0	**1**
0	1	1	**0**
1	0	0	**1**
1	0	1	**0**
1	1	0	**1**
1	1	1	**1**

μ200(t+1) = NOT *Snail(t)* OR *(Zeb(t)* AND *μ200(t)*)

Snail(t)	***Zeb(t)***	***μ200(t)***	***μ200(t+1)***
0	0	0	**1**
0	0	1	**1**
0	1	0	**1**
0	1	1	**1**
1	0	0	**0**
1	0	1	**0**
1	1	0	**0**
1	1	1	**1**

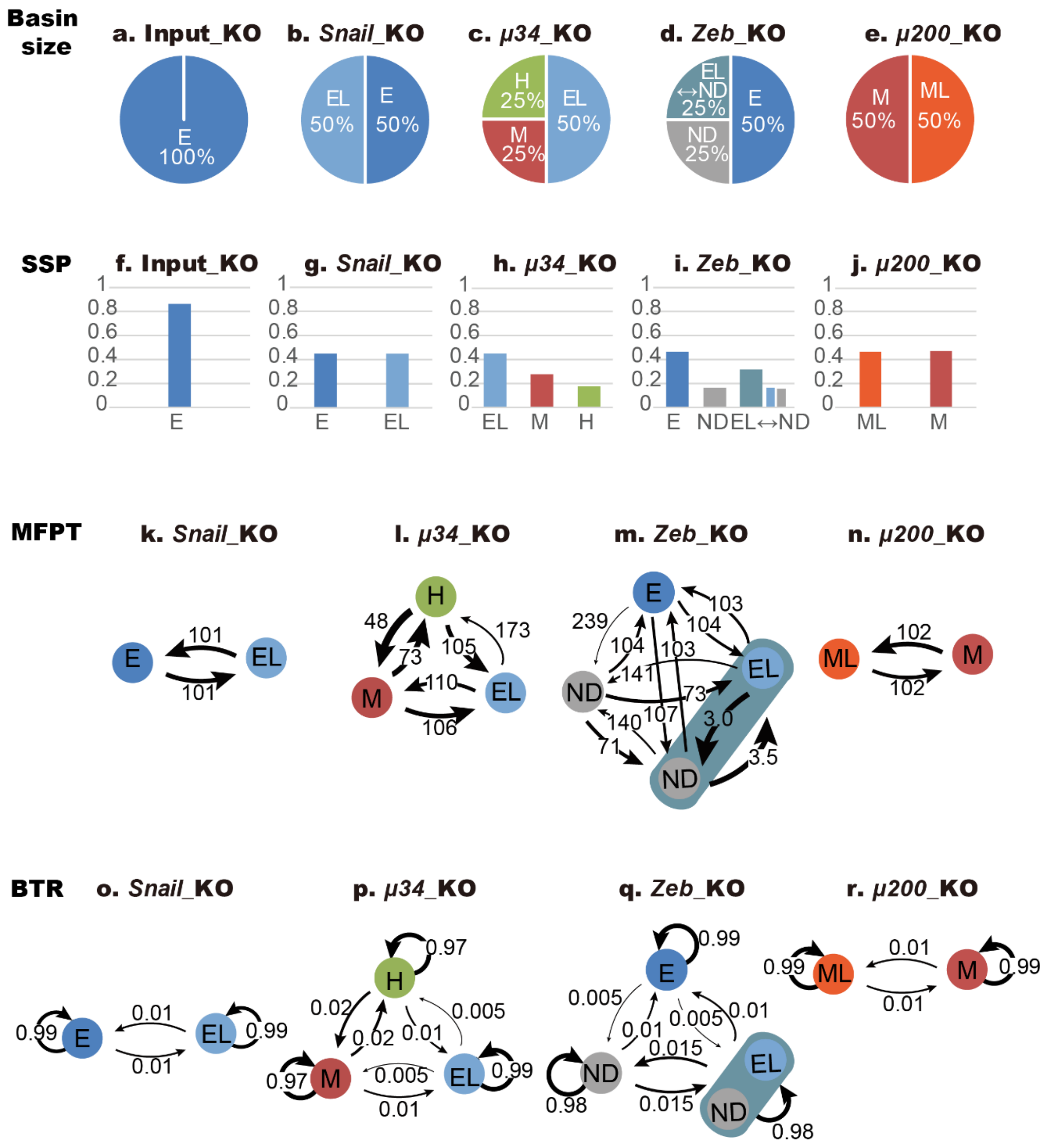

Supplementary Figure S1. **The relative dynamic stabilities of attractors in EMT Boolean model with single KO mutation.**

(**a-e**) The attracting basin size ratio of three attractors for each single gene knock-out. (**f-j**) The steady state probability distribution of three attractors for each single gene knock-out. For cyclic attractors, narrow bars represent steady state probability distribution of each state consisting the cyclic attractor and thick bar represent summed probability of them. (**k-n**) The mean first passage time between three attractors for each single gene knock-out. (**o-r**) The basin transition rates between three attractors for each single gene knock-out. States of cyclic attractor are grouped within the same color block. Note that we cannot analyze MFPT and BTR of the “Input node knock-out” case because the case had only one cell attractor.

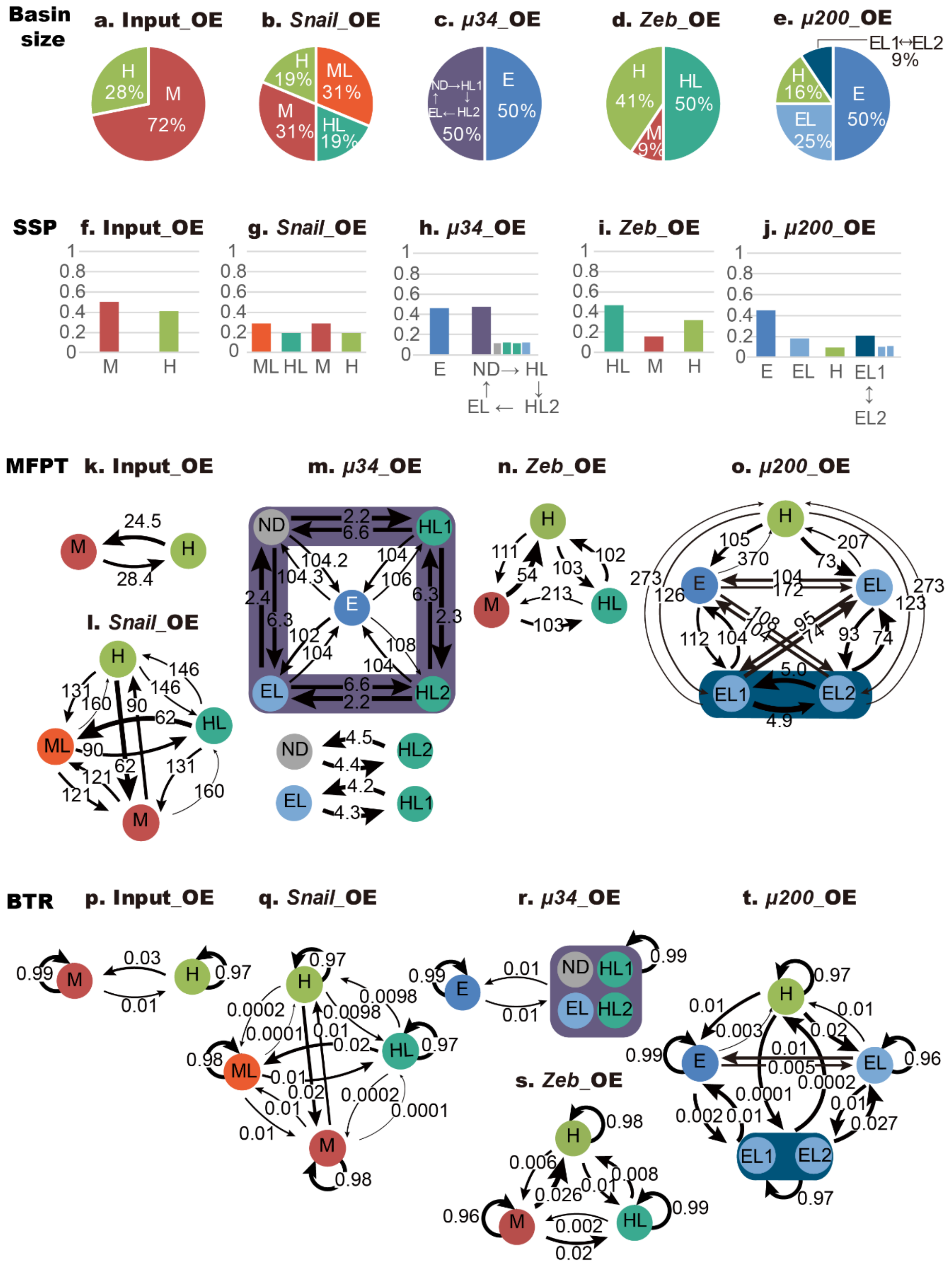

Supplementary Figure S2. **The dynamic stability of EMT Boolean model with a single gene over-expression**

(a-e) The attracting basin size ratio of three attractors for each single gene over-expression. **(f-j)** The steady state probability distribution of three attractors for each single gene over-expression. **(k-n)** The mean first passage time between three attractors for each single gene over-expression. **(o-r)** The basin

transition rates between three attractors for each single gene over-expression. States of cyclic attractor are grouped within one color block.

Supplementary File. SBML-qual format of EMT Boolean model (sbml).

Supplementary Table S2. Data for the relative dynamic stability of EMT Boolean model with single mutation (xlsx).

Supplementary Table S3. 1-degree neighbor of wild type and mutated EMT network (xlsx).
